# Left Ventricular Ring-like Pattern: The Arrhythmic Tale of a Scarred Heart

**DOI:** 10.3390/jcdd12070275

**Published:** 2025-07-17

**Authors:** Vanda Parisi, Claudio Bergami, Ferdinando Pasquale, Maria Alessandra Schiavo, Irene Ruotolo, Naomi Fanciullo, Nicolò Sini, Matteo Ziacchi, Mauro Biffi, Raffaello Ditaranto, Maddalena Graziosi, Elena Biagini

**Affiliations:** 1 Cardiology Unit, Cardiac Thoracic and Vascular Department, IRCCS Azienda Ospedaliero-Universitaria di Bologna, Via Giuseppe Massarenti 9, 40138 Bologna, Italy; vandap22@gmail.com (V.P.); claudio.bergami2@studio.unibo.it (C.B.); elena.biagini73@gmail.com (E.B.); 2 Department of Medical and Surgical Sciences (DIMEC), University of Bologna, Via Irnerio 49, 40126 Bologna, Italy; 3 European Reference Network for Rare, Low Prevalence and Complex Diseases of the Heart—ERN GUARD-Heart, Amsterdam, The Netherlands

**Keywords:** cardiac magnetic resonance, CMR, LGE, scar, phenotype, ring-like, aetiology

## Abstract

Cardiac magnetic resonance (CMR) imaging provides significant advantages in the non-invasive diagnosis of cardiac diseases. An emerging phenotype is increasingly being described in CMR reports, the LGE “ring-like” pattern, which resembles a circumferential/semi-circumferential LV scar. Different conditions exhibit this fibrosis distribution, the majority of them being genetically determined and mostly involving cardiomyopathy-causative genes (desmosomal but also other non-desmosomal related genes). Furthermore, inflammatory diseases, such as myocarditis or sarcoidosis, could be responsible for LV fibrosis, potentially exhibiting an RL distribution. Given the heterogeneity of such conditions, effective patient management requires a stepwise and multiparametric diagnostic work-up that integrates clinical, instrumental, and genetic data to identify the specific aetiology and guide personalised treatments.

## 1. The Ring-like (RL) Scar: Definition

Over the past decade, cardiac magnetic resonance (CMR) imaging has experienced rapid growth thanks to its non-invasive and radiation-free ability to provide a detailed cardiac assessment. It represents the gold standard for evaluating patients with known or suspected cardiomyopathies, providing morphological and functional description as well as tissue characterisation with mapping imaging and late gadolinium enhancement (LGE) [1,2]. The pivotal role of CMR in non-ischemic cardiomyopathies is emphasised by the 2023 European Society of Cardiology (ESC) Guidelines on Cardiomyopathies, which recommend a multimodality and multiparametric approach for both diagnostic and management strategies [3].

**Figure 1:**
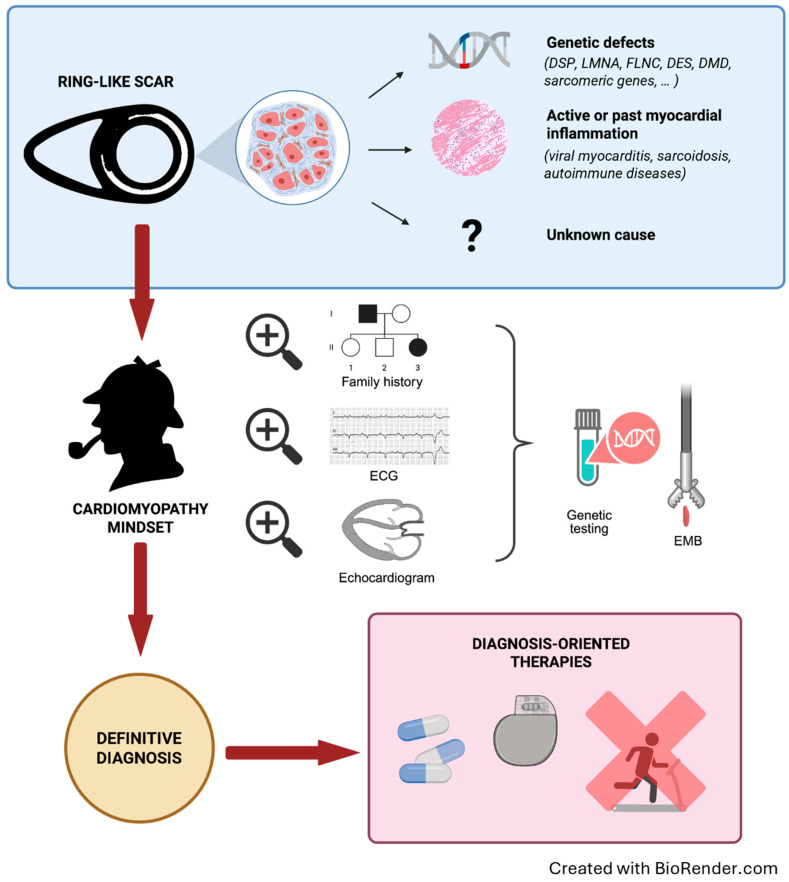
Multiparametric and stepwise management of patients with a RL scar. **Step 1.** Be aware of the ring-like scarring pattern and the possible underlying causes. **Step 2.** A multimodal and comprehensive approach is required, within a cardiomyopathy-oriented mindset looking for “red-flags” of specific aetiologies. First-level tests include family and personal history, physical examination, ECG, echocardiogram, and laboratory tests. Genetic testing using extensive panels of cardiomyopathy-associated genes is recommended for all patients. Pursue further tests, including endomyocardial biopsy in selected cases. **Step 3.** The definite diagnosis will guide the personalised therapy. *DES*: desmin; *DMD*: dystrophin; *DSP*: desmoplakin; ECG: electrocardiogram; EMB: endomyocardial biopsy; FLNC: filamin C; LMNA: lamin A/C; RL: ring-like.

Non-ischemic LGE is observed in a variety of cardiac conditions, including genetically determined diseases, inflammatory processes, metabolic syndromes, toxic exposures, and systemic disorders. It is generally considered a marker of irreversible myocardial fibrosis, although, in many conditions, such as myocarditis, LGE could represent the interstitial space expansion by oedema in conjunction with inflammatory cell infiltration [4]. Among non-ischemic LGE a “ring-like” (RL) pattern is defined as subepicardial/intramyocardial LGE spanning ≥3 contiguous left ventricular (LV) segments in a single short-axis plan. This entity has gained particular attention during recent years and is increasingly being described in CMR reports among the most varied scenarios (Figure 1) [5].

## 2. Unlocking the RL Scar: A CMR Insight into Multiple Conditions

### 2.1. Genetic Cardiomyopathies

Myocardial scars detected by LGE are found in 25–30% of patients with dilated cardiomyopathy (DCM) and hold independent prognostic value beyond left ventricular ejection fraction (LVEF) in the prediction of sudden cardiac death (SCD) [6,7]. Notably, mid-wall/sub-epicardial LGE has been associated with a higher incidence of ventricular arrhythmias (VAs) even in patients with preserved or mildly reduced LVEF, while those with severely reduced LVEF but no LGE may have a lower arrhythmic burden [8,9]. LGE presence and characteristics, such as extension, location, and distribution pattern, are crucial determinants of SCD risk [9,10,11,12,13].

The characteristics of myocardial fibrosis, in conjunction with the genetic background, contribute to the determination of the patient’s phenotype and modulate the associated arrhythmic risk. Studying in detail 89 genotyped patients with DCM, Augusto et al. found an RL-LGE (the “ring of fibrosis”) in 78% of patients harbouring desmoplakin (*DSP*) or filamin-C (*FLNC*) pathogenic/likely pathogenic (P/LP) variants, while the same pattern was absent in other genotypes, including titin (*TTN*) and sarcomeric genes [14]. Segments frequently involved with the RL scar were the basal and medial infero-lateral, characteristically with a subepicardial distribution [14].

In DSP-cardiomyopathy, the pathophysiological process exhibits a preferential involvement of the left ventricle (LV) over the right ventricle (RV), characterised by extensive fibro-fatty replacement within the LV myocardium and potentially associated with subtle LV systolic dysfunction [15]. In an early CMR study, Sen-Chowdhry et al. described a circumferential or near-circumferential LGE “band” in 20% of their genotyped patients (mostly *DSP*) [16]. Similarly, 74% of the *DSP* patients studied by Smith et al. exhibited LV-LGE, 25% of them with a subepicardial circumferential distribution [17]. Specifically, when comparing *DSP* to other desmosomal variants, Laredo et al. found RL-LGE almost exclusively in the *DSP* group (43% vs. 3%, *p* < 0.0001), leading to a specificity of 97% in their cohort [18].

In one of the first genotype–phenotype studies of truncating *FLNC* variants (*FLNCtv*) patients, Ortiz-Genga et al. described the LV fibrosis as “concentric” and circumferential [19]. Likewise, a small cohort of Belgian genotyped patients found a higher prevalence of RL-LGE in *FLNC-tv* than *TTN* (84% vs. 14%, *p* < 0.01) [20]. However, other reports found a lower prevalence of the RL-LGE in *FLNC* patients, only 2/11 (18%) in an Italian population, suggesting a high phenotypic variability [21].

Phospholamban (PLN)-related cardiomyopathy is similarly characterised by widespread myocardial fibro-adipose infiltration. In a cohort of 150 carriers of the *PLN* p.Arg14del variant, a founder variant in the Netherlands, LV-LGE was observed in 33% of the patients, most frequently but not exclusively in those with LVEF < 45%, and mainly in the inferolateral wall with a distribution that resembles the RL pattern [22]. However, other infrequent *PLN* variants may lead to unusual manifestations and evolving phenotypes [23].

Furthermore, a subepicardial and “annular” pattern of LV fibrosis was described in desmin (*DES*) patients, and this circumferential distribution was more frequent than the other LGE patterns observed in desmosomal/negative-genotype subjects in the study from Segura-Rodriguez et al. [24]. More recently, a multicentre study from 10 European centres specifically analysed *DES*-related cardiomyopathy, including 82 patients from 16 families, 49% of them expressing a subepicardial LV-RL scar [25].

However, an RL scar involving the LV could also be found in other genotypes, such as lamin A/C (*LMNA*) or even sarcomeric genes, sometimes with predominant septal involvement [26]. Finally, even in Duchenne/Becker muscular dystrophy patients, the CMR frequently shows LV LGE, starting from the inferolateral wall and spreading through the contiguous LV segments along with the disease progression, frequently in a semi-circumferential fashion that resembles a RL scar [27].

Although our understanding of the molecular basis of desmosomal and desmosomal-related genes has increased during recent decades, the underlying mechanisms responsible for fibro-adiposis in the damaged myocardium remain largely unclear. Disruption of the structural and functional cardiomyocyte integrity, leading to a mechano-chemical failure, has been advocated as the principal mechanism in many of the conditions; however, many signalling pathways still need to be clarified [28]. Moreover, some genetic substrates are linked to very dissimilar phenotypic expressions. Indeed, while many *DES*-related cardiomyopathies are phenotypically comparable to *DSP* and *FLNC* (specifically truncated *FLNC* mutations), other *DES* mutations are associated with different cardiac expressions, such as restrictive cardiomyopathy with conduction abnormalities and neuromuscular involvement [29,30].

The routine use of CMR in patients with cardiomyopathies, specifically DCM and arrhythmogenic right ventricular cardiomyopathy (ARVC), along with the increase in genetic insights, allowed us to understand that many DCM patients are more prone to experiencing arrhythmic events and that, on the other hand, a proportion of ARVC patients also exhibit LV involvement, which could be the main manifestation of the disease [31]. In both cases, an RL scar, with or without LV fatty replacement, could be the primary CMR finding, and the LV is mostly non-dilated (or mildly dilated) with a normal or nearly normal systolic function. These phenotypic similarities have given rise to several classification efforts in the past decade, yet nosological uncertainty persists across the literature. To account for this phenotypic complexity, the 2023 ESC Guidelines on cardiomyopathies introduced the NDLVC phenotype alongside the traditional classifications of DCM and ARVC, emphasising that NDLVC should be regarded solely as an initial morpho-functional descriptor rather than a definitive diagnosis, which must be established through a comprehensive clinical evaluation [3].

Concurrently, a European Task Force consensus report gives a wider definition of “arrhythmogenic cardiomyopathy” (ACM), which is presented in classical ARVC, arrhythmogenic LV cardiomyopathy (ALVC), and biventricular forms [32]. By definition, the RL scar is considered a major criterion and the diagnostic hallmark for ALVC. In addition, the term “scarring-arrhythmogenic cardiomyopathy” was introduced to include non-genetic conditions [33]. It is important to recognise that any classification system remains an attempt to categorise conditions that may differ significantly, each potentially requiring a dedicated clinical approach and carrying different prognostic outcomes.

Prognostic data in patients with an RL-LGE scar harbouring a genetic substrate remain limited and are derived from small or heterogeneous cohorts. In one of the first reports of the RL-LGE pattern, Chen et al. described a cohort of 157 DCM patients who underwent CMR, divided into the following four groups: (i) no LGE; (ii) focal LGE; (iii) multifocal LGE; (iv) RL-LGE [34]. After a median follow-up of 13 ± 7 months, patients with multi-focal/RL-LGE experienced more arrhythmic events than patients with no/focal LGE (25% and 42% vs. 6% and 10%, respectively). After multivariable adjustment, RL-LGE remained associated with an increased risk of VAs, independently of the global LGE burden, while no difference in the incidence of VAs was found between patients with LVEF > 35% and LVEF ≤ 35%. Interestingly, RL-LGE patients showed higher LVEF and smaller left ventricular end-diastolic volume indexed (LVEDVi) [34].

Aiming to evaluate the prognostic utility of LGE in patients with an LV predominant or biventricular disease (labelled as “non-classical ARVC”), Yang et al. found the RL-LGE pattern in 41% of the cohort, and it conferred a higher risk of sustained VAs compared to a non-RL-LGE phenotype or no LGE at all [35]. The RL-LGE was independently associated with the arrhythmic outcome (*p* = 0.036) and, when added to the 2019 ARVC risk model, showed incremental prognostic value for sustained VAs [35,36]. Considering the growing importance of the topic, we conducted a study specifically addressing the characterisation and risk stratification of a multicentric cohort of patients with an RL scar in a “cardiomyopathic scenario” [37]. We enrolled 115 patients with RL-LGE and at least one of the following: (i) a positive genetic test; (ii) a family history of cardiomyopathy; (iii) ACM diagnosis based on the recent 2024 European Task Force consensus criteria [32]. After a median follow-up of 4.6 years, the arrhythmic outcome, composed of SCD, ICD intervention, or sustained VAs, was reached by 19% of patients (3.8 events/100 patient-year). In the multivariate analysis, the presence of anterior Q waves, QRS interval, and LVEDVi at CMR were associated with the outcome. Interestingly, 15% of patients showed a normal ECG and no one among them experienced adverse events, suggesting a possible protective role of a normal ECG [37].

Conversely, a recent study by Gueli et al., which included 225 patients with an NDLVC phenotype and available genetic data, identified RL-LGE in 36% of cases [38]. Despite this, RL-LGE did not emerge as a predictor of the primary arrhythmic endpoint, a finding the authors explained by the limited number of arrhythmic events (5%) recorded over a median follow-up of 3.3 years [38]. As acknowledged by the authors, the absence of prognostic significance of the RL-LGE pattern and genetic markers in their cohort may be explained by the low event rate as well as the limited prevalence of P/LP variants, thereby underscoring the need for larger cohorts to validate these observations [38].

A post hoc analysis of the Cardiac Magnetic Resonance for Primary Prevention Implantable Cardioverter Defibrillator Therapy (DERIVATE) International Registry investigated arrhythmic risk in patients with NDLVC [39]. The prevalence of major adverse arrhythmic events (MAACEs) was 4% in the NDLVC population, compared to 10% in patients with DCM. Patients classified as having “hypokinetic” NDLVC demonstrated significantly lower rates of MAACE compared to those with non-ischemic dilated cardiomyopathy (NIDCM) (*p* = 0.001), while those with “fibrotic” NDLVC showed similar rates for both the primary (*p* = 0.48) and secondary (*p* = 0.616) endpoints compared to the NIDCM group. The prevalence of myocardial fibrosis, particularly mid-wall fibrosis, was a significant predictor of MAACE. In contrast, the presence of combined septal and free-wall LGE, the extension of LGE in >3 segments, and an RL scar were independently associated with all-cause death but not with an increased risk of MAACE [39].

### 2.2. Inflammatory Disorders

Non-ischemic LGE is often found in the context of acute myocarditis and represents a fundamental criterion for the non-invasive CMR diagnosis according to the modified Lake Louise criteria [40]. A multicentre Italian study on 386 patients reported a 93% prevalence of LGE in the setting of acute myocarditis, with an increased risk of heart failure (HF) and VA events when LGE had an anteroseptal mid-wall distribution [41]. Interestingly, a follow-up CMR sub-study on 187 patients found that only 10% showed a disappearance of LGE at 6 months. At the multivariate analysis, the persistence of LGE in the absence of oedema, as well as mid-wall or anteroseptal LGE, was associated with the combined HF-VAs endpoint at the median follow-up of 7 years [42]. Studying 670 patients with suspected acute myocarditis, Gräni et al. found LGE in significantly fewer cases (44%), but its presence was per se associated with major adverse cardiovascular events (MACEs), especially in mid-wall anteroseptal distribution [43].

Recently, a singlecentre Chinese study described an RL-LGE pattern in 14% of patients with an “inflammatory cardiomyopathy”, defined by the presence of heart failure with reduced ejection fraction (HFrEF) after clinically suspected myocarditis [44]. In detail, among the 47 myocarditis patients with an RL-LGE pattern, adverse events were experienced by 14 (31%), while a single event was recognised in the non-RL-LGE group (1/72, 1.4%) [44].

Myocarditis-like episodes, clinically indistinguishable from sporadic and infective ones, could also be one of the clinical manifestations of genetic conditions, such as *DSP* or *FLNC* cardiomyopathies (estimated prevalence of 5–20%) [15,17,45,46]. In a multicentre retrospective study, Ammirati et al. described a population of patients with acute myocarditis and their genetic data, showing that those with a family history of myocarditis, non-sustained VAs, and a septal/RL-LGE were more likely to carry a desmosomal gene variant (mostly *DSP*) (68% and 85% for RL and septal LGE, respectively) [47]. The presence of a desmosomal gene variant was associated with a higher risk of adverse outcomes, including death, VAs, and recurrent episodes of myocarditis (62% at 5-year follow-up) [47]. A recent multicentre study in Catalunya (Spain) evaluated 30 adolescents with infarct-like myocarditis [48]. Genetic testing of 27 patients using a 174-gene panel (excluding *FLNC*) identified P/LP variants in 22%, with a significant correlation to the RL-LGE pattern. Over a median follow-up of 3 years, 9 patients (30%) experienced a myocarditis recurrence, and this happened more frequently in genotype-positive patients who exhibited a greater LGE burden, although the limited sample size reduced the statistical power of the results [48].

Non-ischemic LGE is also observed in other chronic inflammatory conditions, such as cardiac sarcoidosis (CS), in which it represents the delayed contrast washout from the interstitium occupied by granulomatous inflammation and scarring. CMR has become a cornerstone in the non-invasive CS diagnosis. Cine images may reveal reduced LVEF, non-coronary wall motion abnormalities, and basal aneurysms. Moreover, the detection of non-ischemic LGE, typically with patchy and multifocal distribution, is widely recognised not only as a major diagnostic criterion but also as a key prognostic marker [49,50]. In a recent Finnish report of 305 patients with CS (definite diagnosis 45%), 96% had LV-LGE, and, while the most frequent patterns were multifocal LV/septal (defined as “pathology-frequent” distribution of LGE), 40% showed an RL-LGE [51]. This pattern was associated with a high risk of SCD (*p* < 0.001), although the best prediction model resulted from the inclusion of the LGE extension (≥6 LV segments or ≥9.9% of the LV mass) rather than the specific pattern [51].

### 2.3. Unselected Patients Undergoing CMR and Apparently Healthy Individuals

Recently, two large CMR retrospective studies were performed, aiming to analyse specifically the clinical and genetic profiles of an RL-LGE scar found in unselected patients undergoing CMR. Filomena et al. reported a 0.77% prevalence of RL patterns among 23,470 patients referred for a CMR scan (1.1% prevalence of CMR with LGE analysis) [52], with preferential involvement of the inferolateral segments and a median of 10 LV-involved segments. These 152 patients with RL-LGE expressed different morpho-functional phenotypes, mostly DCM and NDLVC, but ARVC and hypertrophic cardiomyopathy (HCM) were also found. Genetic testing was performed in 66% of the cohort and revealed a P/LP variant in 58% of the genotyped patients, including both desmosomal (*DSP* and desmoglein 2–*DSG2*) and non-desmosomal genes (*FLNC*, *DES*, lamin A/C–*LMNA*, *PLN*, *TTN,* and sarcomeric genes). Almost 16% had an inflammatory cardiomyopathy, and there were sporadic cases of rare etiologies such as genetic neuromuscular diseases and inborn errors of metabolism [52]. After a median follow-up of 3 years, the primary composite endpoint (all-cause mortality, left ventricular assist device (LVAD) implantation, and heart transplantation) occurred in 18% of patients. Moreover, 17% of patients experienced arrhythmic events, including sustained VAs, ICD interventions, and SCD [52], which were independently predicted by male sex and RV disproportion (ratio between LV and RV end-diastolic volumes <0.9) but not by LVEF, genetic status, or LGE extension/location.

Similarly, Bietenbeck et al. found a 1.16% prevalence of RL-LGE in 3253 patients referred for CMR. Genetic testing showed a P/LP variant in 61% of cases, mostly *FLNC*, *LMNA,* and desmosomal genes [53]. Among the 38 RL-LGE patients, 63% experienced VAs (both sustained and non-sustained). Interestingly, the following subtle but remarkable differences were evidenced between the different causes of RL-LGE: *FLNC* expressed a more complete mid-wall RL-LGE; desmosomal-RL had more extensive and subepicardial LGE, usually located at the inferolateral segments with less frequent septal involvement; myocarditis showed a more patchy involvement; and *LMNA*-RL was mostly located in the basal septal segments with an intramural pattern [53].

Lastly, it is worth mentioning that several studies reported a significant prevalence of myocardial scarring on CMR in apparently normal phenotypes, including patients with idiopathic premature ventricular complexes (PVCs) traditionally associated with good outcomes [54]. Muser et al. performed CMR on 518 patients with >1000 PVC/24 h and a negative routine work-up, including athletes identified at pre-participation screening [55]. They found LGE in 16% of the cohort, of whom 29% expressed RL-LGE. After a follow-up of 67 months, 29% of patients with LGE experienced arrhythmic events vs. 0.2% of the patients without LGE. Considering only patients with RL-LGE, 57% experienced adverse events, including all 3 SCD cases of the cohort [55].

## 3. RL Scar: From the CMR Findings to Personalised Patient Care

Considering all the conundrums of conditions potentially expressed with an RL scar, the suggested approach is grounded in comprehensive multidisciplinary patient management [56]. The aim is to understand if the RL-LGE, found on a CMR performed for any reason, is the expression of a disease that carries a high risk of SCD, either genetic or acquired (Figure 1).

**Figure 2:**
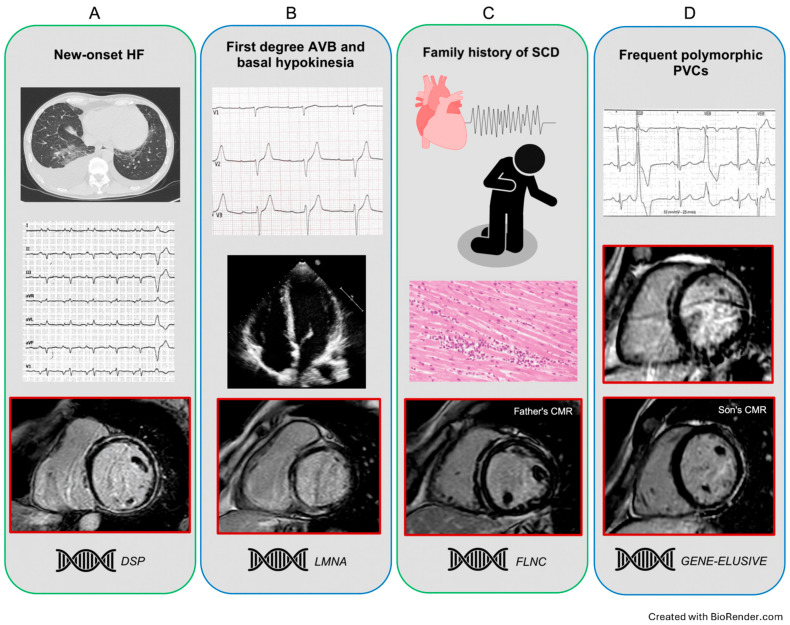
Representative clinical scenarios exhibiting a ring-like pattern. Panel (**A**) A middle-aged man without previous clinical history, presenting for HF symptoms, with severe LV dilatation and systolic dysfunction. Note the low limb leads QRS voltages and the circumferential LGE scarring at CMR. The genetic testing identified a DSP pathogenic variant. Panel (**B**) Asymptomatic young woman with first-degree AV block and LV basal hypokinesia, with normal volumes and EF. The CMR showed an RL-LGE pattern with predominant septal involvement; a pathogenic LMNA variant was identified. Panel (**C**) A middle-aged man with a LV-RL scar was diagnosed within the cardiac screening after his 10-year-old son’s SCD at rest and post-mortem identification of severe biventricular myocarditis. The subsequent genetic analysis revealed a FLNC pathogenic variant in both the proband and his father. Panel (**D**) A middle-aged woman with a long history of apparent idiopathic polymorphic PVCs and an RL-LGE. Although a negative genetic test, the subsequent family screening identified a RL scarring pattern in her asymptomatic son. AV: atrioventricular; CMR: cardiac magnetic resonance; DSP: desmoplakin; EF: ejection fraction; FLNC: filamin C; HF: heart failure; LGE: late gadolinium enhancement; LMNA: lamin A/C; LV: left ventricle; PVCs: premature ventricular contractions; RL: ring-like.

Adopting a “cardiomyopathy-oriented mindset” is imperative, searching first for a family history of cardiomyopathy, cardiac transplant, ICD or pacemaker (PM) implantation, or SCD, but also myocarditis and muscular diseases/symptoms. In any case, it is of paramount importance to actively perform a family screening, including at least first-degree relatives. Notably, an RL-LGE could be identified in patients with a completely normal ECG and echocardiogram, so family members need to also undergo CMR (Figure 2) [16,37,45].

The ECG analysis may reveal low QRS voltages and/or negative T waves (typically in infero-lateral leads) in most desmosomal variants, while conduction delays and atrioventricular blocks (AVBs) are observed in *LMNA* cases [15,45,57]. Cardiomyopathies due to dystrophin (*DMD*) variants classically mimic a posterior, inferior, and/or lateral myocardial infarction, with abnormal Q waves in leads I, aVL, and V6, or in leads II, III, and aVF, together with high-voltage R waves in leads V1 and V2 [58]. ECG findings in cardiac sarcoidosis include conduction delay, AVB, QRS fragmentation, and bundle-branch blocks [59].

Cutaneous signs of desmosomal-related cardiomyopathies and neuromuscular symptoms for *FLNC* or *DES* variants should be searched for, and laboratory tests with creatine phosphokinase (CPK), angiotensin-converting enzyme (ACE), and an autoimmunity screening for the exclusion of genocopies and phenocopies are recommended [17,56,60].

The occurrence of chest pain episodes, even in relatives, needs to be considered and investigated carefully in these patients given the existence of so-called “hot phase” episodes in many underlying genetic substrates [46]. Other clinical presentations leading to the recognition of an RL-LGE include sustained VAs, frequent PVCs/NSTV at Holter monitoring, and heart failure (Figure 2) [45]. Echocardiography may show a dilated and hypokinetic LV (DCM phenotype), a non-dilated LV with global or regional systolic dysfunction (NDLVC), and finally, RV involvement in cases suspected of ARVC.

Genetic testing should be considered in all patients with RL-LGE, given the relatively high rate of positive findings (40–60%) reported in previous studies [52,53]. It is vital to understand that a familiar disease could be present even with a negative genetic test, so relatives need to be evaluated in any case. At the same time, a genetic disease may be identified in the absence of a clear family history given the variable penetrance and expressivity characteristic of genetic cardiomyopathies [3,56].

If an inflammatory disease is suspected, total-body Positron Emission Tomography with 2-deoxy-2-[fluorine-18] fluoro-D-glucose (18F-FDG PET), preferably with Computed Tomography (18F-FDG PET/CT), is indicated [50]. Finally, in selected cases, an endomyocardial biopsy could be required [56].

As recommended by the 2023 ESC Guidelines on Cardiomyopathies, the final goal is to reach the etiological diagnosis behind any phenotype, most commonly DCM and NLDVC. With the suggested “red-flag” approach, the probability of detailing the nature of the underlying condition is as high as possible, with consequently personalised risk strategies [3]. Of note, although awareness and attention to cardiomyopathies have increased over the past few years, a significant proportion of patients still experience substantial diagnostic delays, impacting prognosis as well as cardiologic screening for relatives [61].

The effect of drug therapy is poorly studied in LV-RL patients. In the case of clinical HF, medical therapy should follow specific guidelines and indications [3]. For patients with reduced EF (HFrEF), the cornerstone of treatment includes angiotensin receptor–neprilysin inhibitors (ARNIs), angiotensin-converting enzyme inhibitors (ACEIs)/angiotensin receptor blockers (ARBs), beta-blockers, mineralocorticoid receptor antagonists (MRAs), and sodium-glucose cotransporter-2 (SGLT2) inhibitors, as well as newer agents like vericiguat and finerenone [62,63]. These therapies have been shown to reduce all-cause mortality and HF-related hospitalisations. In patients with mildly reduced ejection fraction (HFmrEF) and those with preserved ejection fraction (HFpEF), SGLT2 inhibitors and MRAs have shown benefit in reducing morbidity and mortality, with increasing evidence supporting the role of finerenone and vericiguat in selected populations. Treatment should be individualised based on clinical profile, comorbidities, and tolerability [62,63].

Anti-arrhythmic drugs (AADs) are traditionally prescribed to prevent arrhythmias in many cardiomyopathies. Guidelines suggest beta blockers (BBs) in case of a defined ARVC diagnosis (class IIB) or in the presence of PVC or VAs (class IIA) [3]. In case of arrhythmia recurrence, flecainide or amiodarone may be added, and catheter ablation could be considered in specialised centres (class IIA). In DCM and NDLVC, BBs should be started when EF < 40% (or <50%, class IIB) for HF prevention; in the case of VAs, the addition of amiodarone, sotalol, or catheter ablation should be considered [3]. In the absence of data in RL patients, it is not clear when BBs should be prescribed in the absence of EF reduction or VAs.

Periodic outpatient visits are recommended, including ECG and echocardiography. Annual monitoring with Holter–ECG or implantable loop recorders needs to be performed in every patient to define the arrhythmic burden, since the identification of NSVT or frequent PVCs is associated with an increased risk of adverse events in several diseases [3,36]. Follow-up CMR may be considered in patients without an ICD to evaluate LGE expansion, which could be associated with worse outcomes [64].

Indications for SCD risk stratification follow the recommendations of the determined RL-LGE aetiology. For patients in the DCM and NDLVC phenotype, a class IIA for primary prevention ICD implantation is reserved for patients with LVEF < 35% or with a high-risk gene variant. In the absence of the aforementioned risk factors, the class of recommendations is lowered to IIB, and the decision to implant an ICD must be weighed carefully, balancing the benefit of protection from SCD with the well-known risk of device-related complications [3]. Frequent NSVT, syncope, family history of SCD, and an extensive amount of fibrosis are additional recognised risk factors for worse outcomes [3]. It is worth noting that for some genotypes, such as *LMNA*, *DSP*, *PLN*, and *FLNC*, dedicated risk calculators are available [65,66,67,68]. Decisions for ICD implantation in patients with a final diagnosis of cardiac sarcoidosis should be managed according to the most recent expert recommendations [50]. Secondary prevention ICD implantation is recommended after aborted SCD and in the majority of sustained poorly tolerated VAs, unless a temporary trigger, such as an acute inflammatory episode, is considered responsible for the event [3]. It should be noted that expert consensus is based on observational data rather than randomised controlled trials; hence, the strength of recommendations cannot be overemphasised.

Considering the paucity of clinical long-term data on RL pattern, sports activity indications should be established according to the definitive diagnosis. The 2020 ESC Guidelines on sports cardiology and exercise in patients with cardiovascular disease give a class III recommendation for participation in competitive and high-intensity physical activity in ARVC and DCM patients with extensive LGE [69]. Moderate-intensity recreational activity can be allowed on an individualised approach.

## 4. Future Directions

A deeper understanding of the ring-like (RL) pattern is essential to improve clinical management and refine risk stratification. Artificial intelligence (AI) techniques, particularly machine learning and deep learning, offer valuable support to clinicians by enhancing image analysis through improved precision, reproducibility, and automation. These technologies allow detection of subtle changes, promote standardisation, reduce expert workload, and enable automated quantification of LGE and additional fibrosis markers, such as T1 mapping and extracellular volume (ECV), across myocardial segments [70]. Beyond imaging, AI can identify patients and predict outcomes by recognising complex patterns in electronic health records [71]. Nonetheless, further research is required to fully establish the clinical utility and impact of AI-driven approaches. Finally, large and prospective studies are needed to validate the prognostic significance of the RL scar in predicting arrhythmic events, while accounting for potential confounders such as LVEF and total scar burden.

## 5. Conclusions

The “RL scar” is a recently described CMR phenotype that encompasses several different aetiologies. Although frequently related to a specific genetic substrate, mainly desmosomal and less commonly non-desmosomal genes, it is frequently observed in the absence of known genetic abnormalities. The topography of myocardial involvement may hint at specific disease aetiologies, but definite diagnosis is strictly dependent on a multiparametric approach including patients’ and family history, ECG findings, other imaging techniques, and genetic evaluation (Figure 1). Heightened attention is paid to the risk of VAs, whose prevalence is not negligible, also in the earliest stages of the disease and may be as high as 3.8% yearly when more factors cluster together [37]. Therefore, careful evaluation is needed to appropriately risk-stratify these patients to avoid unnecessary exposure to lifelong ICD therapy, especially in young patients. Medical treatment is also challenging in this scenario, owing to the absence of dedicated studies and to the incomplete understanding of the current guideline-directed medical therapy’s effect in this specific pathologic milieu.

## Data Availability

Not applicable.

## References

[1] Merlo M., Gagno G., Baritussio A., Bauce B., Biagini E., Canepa M., Cipriani A., Castelletti S., Dellegrottaglie S., Guaricci A.I. (2022). Clinical application of CMR in cardiomyopathies: Evolving concepts and techniques: A position paper of myocardial and pericardial diseases and cardiac magnetic resonance working groups of Italian society of cardiology. Heart Fail. Rev..

[2] Limongelli G., Adorisio R., Baggio C., Bauce B., Biagini E., Castelletti S., Favilli S., Imazio M., Lioncino M., Merlo M. (2022). Diagnosis and Management of Rare Cardiomyopathies in Adult and Paediatric Patients. A Position Paper of the Italian Society of Cardiology (SIC) and Italian Society of Paediatric Cardiology (SICP). Int. J. Cardiol..

[3] Arbelo E., Protonotarios A., Gimeno J.R., Arbustini E., Barriales-Villa R., Basso C., Bezzina C.R., Biagini E., Blom N.A., de Boer R.A. (2023). 2023 ESC Guidelines for the management of cardiomyopathies. Eur. Heart J..

[4] Aquaro G.D., De Gori C., Faggioni L., Parisella M.L., Cioni D., Lencioni R., Neri E. (2023). Diagnostic and prognostic role of late gadolinium enhancement in cardiomyopathies. Eur. Heart J. Suppl..

[5] Vaz A., Morales K.R.D.P., Fonseca E.K.U.N., Souza J.P.S., Rahal M.J.S., Young L.M., Pereira L.M., Scoppetta L.R.P.D., Filho J.R.P. (2025). Ring-like late gadolinium enhancement: Differential diagnosis and mimics. Radiol. Bras..

[6] Di Marco A., Brown P.F., Bradley J., Nucifora G., Claver E., de Frutos F., Dallaglio P.D., Comin-Colet J., Anguera I., Miller C.A. (2021). Improved Risk Stratification for Ventricular Arrhythmias and Sudden Death in Patients with Nonischemic Dilated Cardiomyopathy. J. Am. Coll. Cardiol..

[7] Brown P.F., Miller C., Di Marco A., Schmitt M. (2019). Towards cardiac MRI based risk stratification in idiopathic dilated cardiomyopathy. Heart.

[8] Halliday B.P., Gulati A., Ali A., Guha K., Newsome S., Arzanauskaite M., Vassiliou V.S., Lota A., Izgi C., Tayal U. (2017). Association between midwall late gadolinium enhancement and sudden cardiac death in patients with dilated cardiomyopathy and mild and moderate left ventricular systolic dysfunction. Circulation.

[9] Halliday B.P., Baksi A.J., Gulati A., Ali A., Newsome S., Izgi C., Arzanauskaite M., Lota A., Tayal U., Vassiliou V.S. (2019). Outcome in Dilated Cardiomyopathy Related to the Extent, Location, and Pattern of Late Gadolinium Enhancement. JACC Cardiovasc. Imaging.

[10] Halliday B.P. (2022). State of the art: Multimodality imaging in dilated cardiomyopathy. Heart.

[11] Balaban G., Halliday B.P., Porter B., Bai W., Nygåard S., Owen R., Hatipoglu S., Ferreira N.D., Izgi C., Tayal U. (2021). Late-Gadolinium Enhancement Interface Area and Electrophysiological Simulations Predict Arrhythmic Events in Patients with Nonischemic Dilated Cardiomyopathy. JACC Clin. Electrophysiol..

[12] Leyva F., Zegard A., Acquaye E., Gubran C., Taylor R., Foley P.W., Umar F., Patel K., Panting J., Marshall H. (2017). Outcomes of Cardiac Resynchronization Therapy with or Without Defibrillation in Patients with Nonischemic Cardiomyopathy. J. Am. Coll. Cardiol..

[13] Piers S.R., Everaerts K., van der Geest R.J., Hazebroek M.R., Siebelink H.-M., Pison L.A., Schalij M.J., Bekkers S.C., Heymans S., Zeppenfeld K. (2015). Myocardial scar predicts monomorphic ventricular tachycardia but not polymorphic ventricular tachycardia or ventricular fibrillation in nonischemic dilated cardiomyopathy. Heart Rhythm.

[14] Augusto J.B., Eiros R., Nakou E., Moura-Ferreira S., Treibel T.A., Captur G., Akhtar M.M., Protonotarios A., Gossios T.D., Savvatis K. (2020). Dilated cardiomyopathy and arrhythmogenic left ventricular cardiomyopathy: A comprehensive genotype-imaging phenotype study. Eur. Heart J. Cardiovasc. Imaging.

[15] Gasperetti A., Carrick R.T., Protonotarios A., Murray B., Laredo M., van der Schaaf I., Lekanne R.H., Syrris P., Cannie D., Tichnell C. (2025). Clinical features and outcomes in carriers of pathogenic desmoplakin variants. Eur. Heart J..

[16] Sen-Chowdhry S., Syrris P., Prasad S.K., Hughes S.E., Merrifield R., Ward D., Pennell D.J., McKenna W.J. (2008). Left-Dominant Arrhythmogenic Cardiomyopathy: An Under-Recognized Clinical Entity. J. Am. Coll. Cardiol..

[17] Smith E.D., Lakdawala N.K., Papoutsidakis N., Aubert G., Mazzanti A., McCanta A.C., Agarwal P.P., Arscott P., Dellefave-Castillo L.M., Vorovich E.E. (2020). Desmoplakin Cardiomyopathy, a Fibrotic and Inflammatory Form of Cardiomyopathy Distinct from Typical Dilated or Arrhythmogenic Right Ventricular Cardiomyopathy. Circulation.

[18] Laredo M., Charpentier E., Soulez S., Nguyen V., Martino A., Calò L., Ader F., Hermida A., Fressart V., Charron P. (2025). Imaging Features of Desmoplakin Arrhythmogenic Cardiomyopathy: A Comparative Cardiac Magnetic Resonance Study. J. Cardiovasc. Magn. Reson..

[19] Ortiz-Genga M.F., Cuenca S., Ferro M.D., Zorio E., Salgado-Aranda R., Climent V., Padrón-Barthe L., Duro-Aguado I., Jiménez-Jáimez J., Hidalgo-Olivares V.M. (2016). Truncating FLNC Mutations Are Associated with High-Risk Dilated and Arrhythmogenic Cardiomyopathies. J. Am. Coll. Cardiol..

[20] Jacobs J., Van Aelst L., Breckpot J., Corveleyn A., Kuiperi C., Dupont M., Heggermont W., De Vadder K., Willems R., Van Cleemput J. (2023). Tools to differentiate between Filamin C and Titin truncating variant carriers: Value of MRI. Eur. J. Hum. Genet..

[21] Celeghin R., Cipriani A., Bariani R., Marinas M.B., Cason M., Bevilacqua M., De Gaspari M., Rizzo S., Rigato I., Da Pozzo S. (2022). Filamin-C variant-associated cardiomyopathy: A pooled analysis of individual patient data to evaluate the clinical profile and risk of sudden cardiac death. Heart Rhythm.

[22] Rijdt W.P.T., Sande J.N.T., Gorter T.M., van der Zwaag P.A., van Rijsingen I.A., Boekholdt S.M., van Tintelen J.P., van Haelst P.L., Planken R.N., de Boer R.A. (2019). Myocardial fibrosis as an early feature in phospholamban p.Arg14del mutation carriers: Phenotypic insights from cardiovascular magnetic resonance imaging. Eur. Heart J. Cardiovasc. Imaging.

[23] Parisi V., Chiti C., Graziosi M., Pasquale F., Ditaranto R., Minnucci M., Biffi M., Potena L., Girolami F., Baldovini C. (2022). Phospholamban Cardiomyopathy: Unveiling a Distinct Phenotype Through Heart Failure Stages Progression. Circ. Cardiovasc. Imaging.

[24] Segura-Rodríguez D., Bermúdez-Jiménez F.J., Carriel V., López-Fernández S., González-Molina M., Ramírez J.M.O., Fernández-Navarro L., García-Roa M.D., Cabrerizo E.M., Durand-Herrera D. (2020). Myocardial fibrosis in arrhythmogenic cardiomyopathy: A genotype-phenotype correlation study. Eur. Heart J. Cardiovasc. Imaging.

[25] Bermudez-Jimenez F.J., Protonotarios A., García-Hernández S., Asensio A.P., Rampazzo A., Zorio E., Brodehl A., Arias M.A., Macías-Ruiz R., Fernández-Armenta J. (2024). Phenotype and Clinical Outcomes in Desmin-Related Arrhythmogenic Cardiomyopathy. Clin. Electrophysiol..

[26] Kovacs B., Ghannam M., Liang J., Moccoro E., Attili A., Cochet H., Helms A., Latchamsetty R., Jongnarangsin K., Morady F. (2023). Value of genotyping and scar-phenotyping for VT ablation procedures in patients with nonischemic left ventricular cardiomyopathies. J. Cardiovasc. Electrophysiol..

[27] Mavrogeni S. (2015). Cardiac involvement in Duchenne and Becker muscular dystrophy. World J. Cardiol..

[28] Austin K.M., Trembley M.A., Chandler S.F., Sanders S.P., Saffitz J.E., Abrams D.J., Pu W.T. (2019). Molecular mechanisms of arrhythmogenic cardiomyopathy. Nat. Rev. Cardiol..

[29] Brodehl A., Ferrier R.A., Hamilton S.J., Greenway S.C., Brundler M.-A., Yu W., Gibson W.T., McKinnon M.L., McGillivray B., Alvarez N. (2016). Mutations in FLNC are Associated with Familial Restrictive Cardiomyopathy. Hum. Mutat..

[30] Ditaranto R., Caponetti A.G., Ferrara V., Parisi V., Minnucci M., Chiti C., Baldassarre R., Di Nicola F., Bonetti S., Hasan T. (2022). Pediatric Restrictive Cardiomyopathies. Front. Pediatr..

[31] Elliott P.M., Anastasakis A., Asimaki A., Basso C., Bauce B., Brooke M.A., Calkins H., Corrado D., Duru F., Green K.J. (2019). Definition and treatment of arrhythmogenic cardiomyopathy: An updated expert panel report. Eur. J. Heart Fail..

[32] Corrado D., Anastasakis A., Basso C., Bauce B., Blomström-Lundqvist C., Bucciarelli-Ducci C., Cipriani A., De Asmundis C., Gandjbakhch E., Jiménez-Jáimez J. (2024). Proposed diagnostic criteria for arrhythmogenic cardiomyopathy: European Task Force consensus report. Int. J. Cardiol.

[33] Corrado D., Zorzi A., Cipriani A., Bauce B., Bariani R., Brunetti G., Graziano F., De Lazzari M., Mattesi G., Migliore F. (2023). Scarring/arrhythmogenic cardiomyopathy. Eur. Heart J. Suppl..

[34] Chen W., Qian W., Zhang X., Li D., Qian Z., Xu H., Liao S., Chen X., Wang Y., Hou X. (2021). Ring-like late gadolinium enhancement for predicting ventricular tachyarrhythmias in non-ischaemic dilated cardiomyopathy. Eur. Heart J. Cardiovasc. Imaging.

[35] Yang Y., Wei X., Lu G., Xie J., Tan Z., Du Z., Ye W., Xu H., Li X., Liu E. (2023). Ringlike late gadolinium enhancement provides incremental prognostic value in non-classical arrhythmogenic cardiomyopathy. J. Cardiovasc. Magn. Reason..

[36] Cadrin-Tourigny J., Bosman L.P., Nozza A., Wang W., Tadros R., Bhonsale A., Bourfiss M., Fortier A., Lie Ø.H., Saguner A.M. (2022). A new prediction model for ventricular arrhythmias in arrhythmogenic right ventricular cardiomyopathy. Eur. Heart J..

[37] Parisi V., Graziosi M., Lopes L.R., De Luca A., Pasquale F., Tini G., Targetti M., Cueto M.R., Moura A.R., Ditaranto R. (2024). Arrhythmic risk stratification in patients with left ventricular ring-like scar. Eur. J. Prev. Cardiol..

[38] Gueli I.A., Aimo A., Alderotti B., Trimarchi G., Bellisario I., Todiere G., Grigoratos C., De Gori C., Clemente A., Fabiani I. (2025). Arrhythmic risk prediction in non-dilated left ventricular cardiomyopathy: The role of overlap with arrhythmogenic cardiomyopathy. Int. J. Cardiol..

[39] Leo I., Dellegrottaglie S., Scatteia A., Torella D., Abete R., Aquaro G.D., Baggiano A., Barison A., Bogaert J., Calo’ L. (2025). CarDiac magnEtic Resonance for prophylactic Implantable-cardioVerter defibrillAtor ThErapy in Non-Dilated Left Ventricular Cardiomyopathy: A sub-study from the DERIVATE Registry. Eur. Heart J. Cardiovasc. Imaging.

[40] Ferreira V.M., Schulz-Menger J., Holmvang G., Kramer C.M., Carbone I., Sechtem U., Kindermann I., Gutberlet M., Cooper L.T., Liu P. (2018). Cardiovascular Magnetic Resonance in Nonischemic Myocardial Inflammation: Expert Recommendations. J. Am. Coll. Cardiol..

[41] Aquaro G.D., Perfetti M., Camastra G., Monti L., Dellegrottaglie S., Moro C., Pepe A., Todiere G., Lanzillo C., Scatteia A. (2017). Cardiac MR with Late Gadolinium Enhancement in Acute Myocarditis with Preserved Systolic Function: ITAMY Study. J. Am. Coll. Cardiol..

[42] Aquaro G.D., Habtemicael Y.G., Camastra G., Monti L., Dellegrottaglie S., Moro C., Lanzillo C., Scatteia A., Di Roma M., Pontone G. (2019). Prognostic Value of Repeating Cardiac Magnetic Resonance in Patients with Acute Myocarditis. J. Am. Coll. Cardiol..

[43] Gräni C., Eichhorn C., Bière L., Murthy V.L., Agarwal V., Kaneko K., Cuddy S., Aghayev A., Steigner M., Blankstein R. (2017). Prognostic Value of Cardiac Magnetic Resonance Tissue Characterization in Risk Stratifying Patients with Suspected Myocarditis. J. Am. Coll. Cardiol..

[44] Wang H., Bo K., Gao Y., Zhou Z., Xu L. (2023). Prognosis evaluation of chronic inflammatory cardiomyopathy with ring-like late gadolinium enhancement. ESC Heart Fail..

[45] Graziosi M., Ditaranto R., Rapezzi C., Pasquale F., Lovato L., Leone O., Parisi V., Potena L., Ferrara V., Minnucci M. (2022). Clinical presentations leading to arrhythmogenic left ventricular cardiomyopathy. Open Heart.

[46] Bariani R., Rigato I., Cipriani A., Marinas M.B., Celeghin R., Basso C., Corrado D., Pilichou K., Bauce B. (2022). Myocarditis-like Episodes in Patients with Arrhythmogenic Cardiomyopathy: A Systematic Review on the So-Called Hot-Phase of the Disease. Biomolecules.

[47] Ammirati E., Raimondi F., Piriou N., Infirri L.S., Mohiddin S.A., Mazzanti A., Shenoy C., Cavallari U.A., Imazio M., Aquaro G.D. (2022). Acute Myocarditis Associated with Desmosomal Gene Variants. JACC Heart Fail..

[48] Esmel-Vilomara R., Riaza L., Dolader P., Rodríguez-Santiago B., Lasa-Aranzasti A., Muñoz-Cabello P., Fernández-Álvarez P., Figueras-Coll M., Bianco L., Bueno-Gómez A. (2025). Infarct-like myocarditis in adolescents: Exploring genetic insights from diagnosis through follow-up. Int. J. Cardiol..

[49] Lehtonen J., Uusitalo V., Pöyhönen P., Mäyränpää M.I., Kupari M. (2023). Cardiac sarcoidosis: Phenotypes, diagnosis, treatment, and prognosis. Eur. Heart J..

[50] Cheng R.K., Kittleson M.M., Beavers C.J., Birnie D.H., Blankstein R., Bravo P.E., Gilotra N.A., Judson M.A., Patton K.K., Rose-Bovino L. (2024). Diagnosis and Management of Cardiac Sarcoidosis: A Scientific Statement from the American Heart Association. Circulation.

[51] Pöyhönen P., Lehtonen J., Syväranta S., Velikanova D., Mälkönen H., Simonen P., Nordenswan H.-K., Uusitalo V., Vihinen T., Kaikkonen K. (2024). Magnetic Resonance Imaging in the Assessment of the Risk of Sudden Death in Cardiac Sarcoidosis: What Is Extensive or Significant Late Gadolinium Enhancement?. Circ. Arrhythmia Electrophysiol..

[52] Filomena D., Vandenberk B., Dresselaers T., Willems R., Masci P.G., Robyns T., Bogaert J. (2025). Cardiac Diagnoses and Long-Term Outcomes in Ring-Like Late Gadolinium Enhancement Evaluated by Cardiac Magnetic Resonance. Eur. Heart J. Cardiovasc. Imaging.

[53] Bietenbeck M., Meier C., Korthals D., Theofanidou M., Stalling P., Dittmann S., Schulze-Bahr E., Eckardt L., Yilmaz A. (2024). Possible Causes and Clinical Relevance of a ‘Ring-Like’ Late Gadolinium Enhancement Pattern. JACC Cardiovasc. Imaging.

[54] Muser D., Santangeli P., Castro S.A., Arroyo R.C., Maeda S., Benhayon D.A., Liuba I., Liang J.J., Sadek M.M., Chahal A. (2020). Risk Stratification of Patients with Apparently Idiopathic Premature Ventricular Contractions: A Multicenter International CMR Registry. JACC Clin. Electrophysiol..

[55] Muser D., Nucifora G., Pieroni M., Castro S.A., Arroyo R.C., Maeda S., Benhayon D.A., Liuba I., Sadek M., Magnani S. (2021). Prognostic Value of Nonischemic Ringlike Left Ventricular Scar in Patients with Apparently Idiopathic Nonsustained Ventricular Arrhythmias. Circulation.

[56] Rapezzi C., Arbustini E., Caforio A.L.P., Charron P., Gimeno-Blanes J., Heliö T., Linhart A., Mogensen J., Pinto Y., Ristic A. (2013). Diagnostic work-up in cardiomyopathies: Bridging the gap between clinical phenotypes and final diagnosis. A position statement from the ESC Working Group on Myocardial and Pericardial Diseases. Eur. Heart J..

[57] Ollila L., Nikus K., Holmström M., Jalanko M., Jurkko R., Kaartinen M., Koskenvuo J., Kuusisto J., Kärkkäinen S., Palojoki E. (2017). Clinical disease presentation and ECG characteristics of LMNA mutation carriers. Open Heart.

[58] Finocchiaro G., Merlo M., Sheikh N., De Angelis G., Papadakis M., Olivotto I., Rapezzi C., Carr-White G., Sharma S., Mestroni L. (2020). The electrocardiogram in the diagnosis and management of patients with dilated cardiomyopathy. Eur. J. Heart Fail..

[59] Birnie D.H., Nery P.B., Ha A.C., Beanlands R.S. (2016). Cardiac Sarcoidosis.

[60] Arbustini E., Di Toro A., Giuliani L., Favalli V., Narula N., Grasso M. (2018). Cardiac Phenotypes in Hereditary Muscle Disorders: JACC State-of-the-Art Review. J. Am. Coll. Cardiol..

[61] Tini G., Graziosi M., Musumeci B., Targetti M., Russo D., Parisi V., Argirò A., Ditaranto R., Leone O., Autore C. (2023). Diagnostic delay in arrhythmogenic cardiomyopathy. Eur. J. Prev. Cardiol..

[62] McDonagh T.A., Metra M., Adamo M., Gardner R.S., Baumbach A., Böhm M., Burri H., Butler J., Čelutkienė J., Chioncel O. (2021). 2021 ESC Guidelines for the diagnosis and treatment of acute and chronic heart failure: Developed by the Task Force for the diagnosis and treatment of acute and chronic heart failure of the European Society of Cardiology (ESC) with the special contribution of the Heart Failure Association (HFA) of the ESC. Eur. Heart J..

[63] McDonagh T.A., Metra M., Adamo M., Gardner R.S., Baumbach A., Böhm M., Burri H., Butler J., Čelutkienė J., Chioncel O. (2023). 2023 Focused Update of the 2021 ESC Guidelines for the diagnosis and treatment of acute and chronic heart failure: Developed by the task force for the diagnosis and treatment of acute and chronic heart failure of the European Society of Cardiology (ESC) with the special contribution of the Heart Failure Association (HFA) of the ESC. Eur. Heart J..

[64] Mandawat A., Chattranukulchai P., Mandawat A., Blood A.J., Ambati S., Hayes B., Rehwald W., Kim H.W., Heitner J.F., Shah D.J. (2021). Progression of Myocardial Fibrosis in Nonischemic DCM and Association with Mortality and Heart Failure Outcomes. JACC Cardiovasc. Imaging.

[65] Gigli M., Stolfo D., Barbati G., Graw S., Chen S.N., Merlo M., Medo K., Gregorio C., Ferro M.D., Filamin C Registry Consortium (2025). Arrhythmic Risk Stratification of Carriers of Filamin C Truncating Variants. JAMA Cardiol..

[66] van der Heide M.Y.C., Verstraelen T.E., van Lint F.H.M., Bosman L.P., de Brouwer R., Proost V.M., van Drie E., Taha K., Zwinderman A.H., Dickhoff C. (2024). Long-term reliability of the phospholamban (PLN) p.(Arg14del) risk model in predicting major ventricular arrhythmia: A landmark study. Europace.

[67] Wahbi K., BEN Yaou R., Gandjbakhch E., Anselme F., Gossios T., Lakdawala N.K., Stalens C., Sacher F., Babuty D., Trochu J.-N. (2019). Development and Validation of a New Risk Prediction Score for Life-Threatening Ventricular Tachyarrhythmias in Laminopathies. Circulation.

[68] Carrick R.T., Gasperetti A., Protonotarios A., Murray B., Laredo M., van der Schaaf I., Dooijes D., Syrris P., Cannie D., Tichnell C. (2024). A novel tool for arrhythmic risk stratification in desmoplakin gene variant carriers. Eur. Heart J..

[69] Pelliccia A., Sharma S., Gati S., Bäck M., Börjesson M., Caselli S., Collet J.-P., Corrado D., Drezner J.A., Halle M. (2021). 2020 ESC Guidelines on sports cardiology and exercise in patients with cardiovascular disease. Eur. Heart J..

[70] Zhang Q., Fotaki A., Ghadimi S., Wang Y., Doneva M., Wetzl J., Delfino J.G., O’rEgan D.P., Prieto C., Epstein F.H. (2024). Improving the efficiency and accuracy of cardiovascular magnetic resonance with artificial intelligence—Review of evidence and proposition of a roadmap to clinical translation. J. Cardiovasc. Magn. Reson..

[71] Rajkomar A., Dean J., Kohane I. (2019). Machine Learning in Medicine. N. Engl. J. Med..

